# Transcriptomic and functional screening of weapon formation genes implies significance of cell adhesion molecules and female-biased genes in broad-horned flour beetle

**DOI:** 10.1371/journal.pgen.1011069

**Published:** 2023-12-05

**Authors:** Miyu Sugiyama, Takane Ozawa, Kunihiro Ohta, Kensuke Okada, Teruyuki Niimi, Katsushi Yamaguchi, Shuji Shigenobu, Yasukazu Okada

**Affiliations:** 1 Department of Biological Sciences, Tokyo Metropolitan University, Hachioji, Tokyo, Japan; 2 Department of Life Sciences, The University of Tokyo, Komaba, Tokyo, Japan; 3 Faculty of Environmental, Life, Natural Science and Technology, Okayama University, Tsushima-naka, Okayama, Japan; 4 National Institute for Basic Biology, Nishigonaka, Myodaiji, Okazaki, Japan; 5 Basic Biology Program, The Graduate University for Advanced Studies, SOKENDAI, Nishigonaka, Myodaiji, Okazaki, Japan; Shizuoka University, JAPAN

## Abstract

For understanding the evolutionary mechanism of sexually selected exaggerated traits, it is essential to uncover its molecular basis. By using broad-horned flour beetle that has male-specific exaggerated structures (mandibular horn, head horn and gena enlargement), we investigated the transcriptomic and functional characters of sex-biased genes. Comparative transcriptome of male vs. female prepupal heads elucidated 673 sex-biased genes. Counter-intuitively, majority of them were female-biased (584 genes), and GO enrichment analysis showed cell-adhesion molecules were frequently female-biased. This pattern motivated us to hypothesize that female-biased transcripts (i.e. the transcripts diminished in males) may play a role in outgrowth formation. Potentially, female-biased genes may act as suppressors of weapon structure. In order to test the functionality of female-biased genes, we performed RNAi-mediated functional screening for top 20 female-biased genes and 3 genes in the most enriched GO term (cell-cell adhesion, *fat1/2/3*, *fat4* and *dachsous*). Knockdown of one transcription factor, *zinc finger protein 608 (zfp608)* resulted in the formation of male-like gena in females, supporting the outgrowth suppression function of this gene. Similarly, knockdown of *fat4* induced rudimental, abnormal mandibular horn in female. *fat1/2/3*^RNAi^, *fat4*^RNAi^ and *dachsous*^RNAi^ males exhibited thick and/or short mandibular horns and legs. These cell adhesion molecules are known to regulate tissue growth direction and known to be involved in the weapon formation in Scarabaeoidea beetles. Functional evidence in phylogenetically distant broad-horned flour beetle suggest that cell adhesion genes are repeatedly deployed in the acquisition of outgrowth. In conclusion, this study clarified the overlooked functions of female-biased genes in weapon development.

## Author summary

A diverse range of animals have evolved the male sexual weapons that provide an advantage in male-male competition. Understanding the genetic mechanisms behind the sexually dimorphic expression of these weapons is crucial for comprehending their evolution. However, this area of study remains largely unexplored. By comparing the gene expression in the heads of male and female prepupae, we identified 673 sex-biased genes, with 584 genes being highly expressed in females. Through knockdown experiments targeting 23 female-biased genes, we discovered that four genes play a role in determining the size and expression of male-exaggerated traits. Intriguingly, two of these genes, including a transcription factor and a cell adhesion molecule, were found to suppress weapon development in females, implying that down-regulation of these genes in males enhances weapon development. These findings illuminate the often-overlooked function of female-biased genes in male weapon development and propose the idea that the evolution of weapons involves the down-regulation of certain genes in males.

## Introduction

Sexual selection, that is male-male competition and female choice, often drives evolution of complex exaggerated traits in males, such as antler horns and peacock ornaments [1,2]. Generally, target traits of sexual selection are extremely enlarged in males whereas females usually lack these costly structures, leading them to have striking sexual dimorphism [1,3].

A vast number of beetles have evolved weapons for male-male combats [1,2]. The best-known example is Scarabaeoidea beetles that include Hercules beetles, dung beetles and stag beetles. In beetles other than scarabs, weapons have also repeatedly evolved in several coleopteran Families such as Chrysomelidae, Staphylinidae, Cerambycidae, Ciidae, and Tenebrionidae [4]. One typical form is “horn” that is a rigid outgrowth of the head and/or thoracic body wall [5]. Another form is the overgrowth of appendages such as the mandibles of stag beetles, and the legs of harlequin beetle and frog beetle [4,6,7]. The repeated modifications of various body parts in beetles give us great opportunity to assess the commonality and diversity of the making of these structures. To achieve a comprehensive understanding, it is essential to uncover the genes contributing the making of exaggerated traits and their sexually dimorphic expression [8].

In this decade, several developmental mechanisms were proposed for insect weapons [8–13], including the growth factors such as juvenile hormone (JH), insulin-like peptide (ILP), bone morphogenetic protein (BMP), and cell proliferation and outgrowth regulators including Fat/Hippo signaling genes (e.g. fat, dachsous; [14]) Importantly, the weapon development driven by above genes and/or factors are modified by sex-determination pathway genes (e.g. *doublesex*) and the resulting phenotypes show the sex-specific growth patterns [15–18]. For example, the JH-mediated overgrowth of stag beetle mandible only occurs under the presence of male-specific *doublesex* isoform [18]. These studies, mostly based on the prediction from general insect development and physiology, substantially advanced the molecular understanding of sexual trait exaggeration. However, the specific focuses on the predicted factors may overlook the potentially important, unexpected genes. The non-biased transcriptomic screening is a powerful tool to discover the novel candidates.

The investigation for the sex- and tissue-biased transcripts is an important first step for finding the genes associated with sexual weapons. In Asian rhinoceros beetle, the transcriptomic analysis elucidated approximately 700 to 4000 sex-biased genes in horn primordia [19,20]. In a dung beetle, more than 2500 genes showed sex-biased expression in pupal head and thorax [21]. Nearly 1500 genes were sex-biased in the exaggerated third leg of a water strider [22]. Given that males develop large complex structures, one would expect that male-biased genes account for the weapon overgrowth, and therefore that male-biased genes are more abundant than female-biased genes. However, female-biased genes are more abundant than male-biased genes in rhinoceros beetle and water strider [20,22] and they are even in number in a dung beetle [23]. Such patterns challenge the notion that female development is the “baseline” [23] and raise a question whether the simple addition of male-biased genes can explain the acquisition of male weapon.

**Fig 1:**
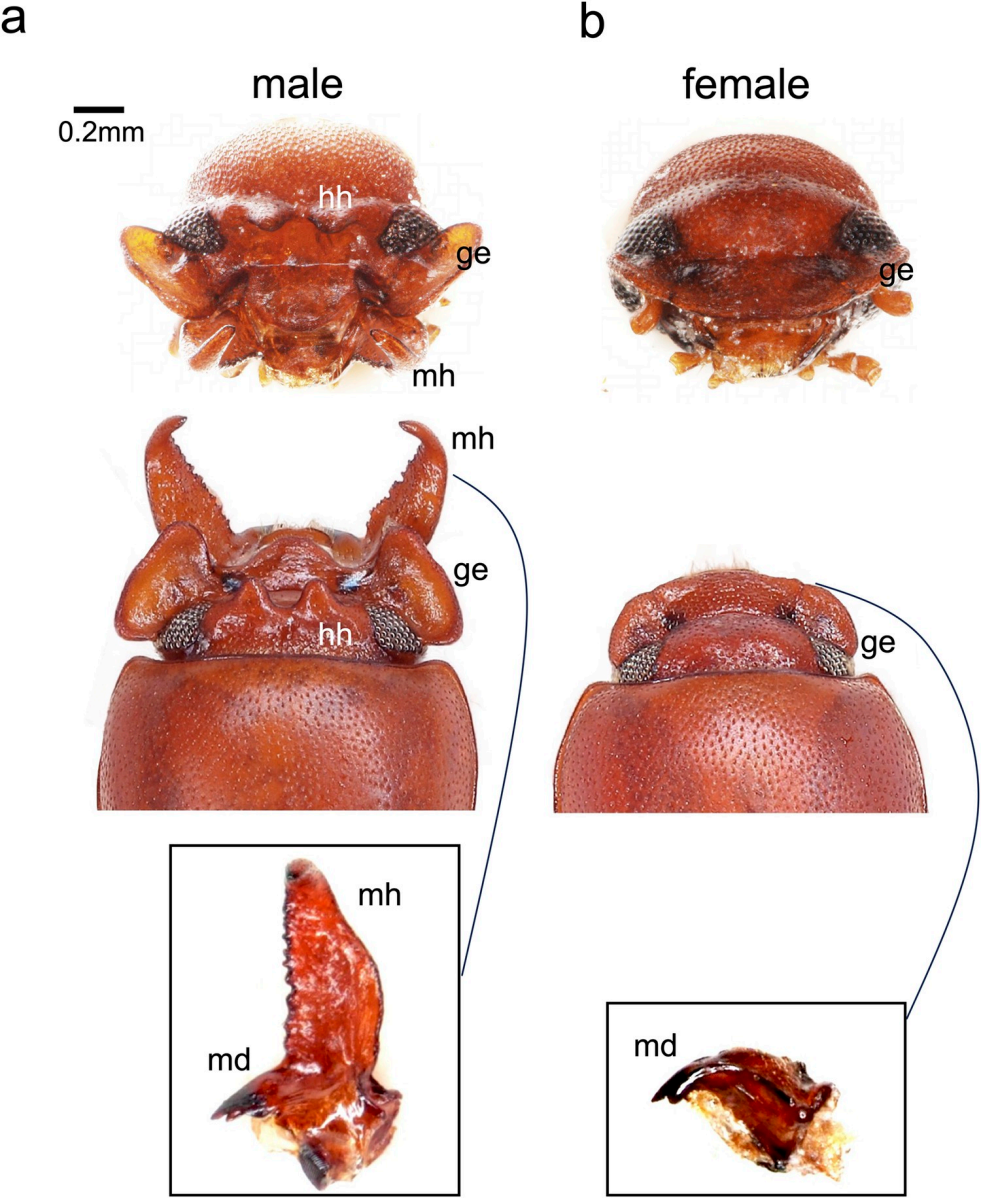
Sexual dimorphism of broad-horned flour beetle (*Gnatocerus cornutus*). a) male, b) female, top, frontal view; middle, dorsal view; bottom (inset), dorsal view of magnified mandibles. Lateral region of male head (gena, ge) is enlarged, and two head horns (hh) are located between eyes (top and middle panels). Females lack mandibular horn, head horn, and lateral protrusion of gena (b, top to bottom panels).

**Fig 2:**
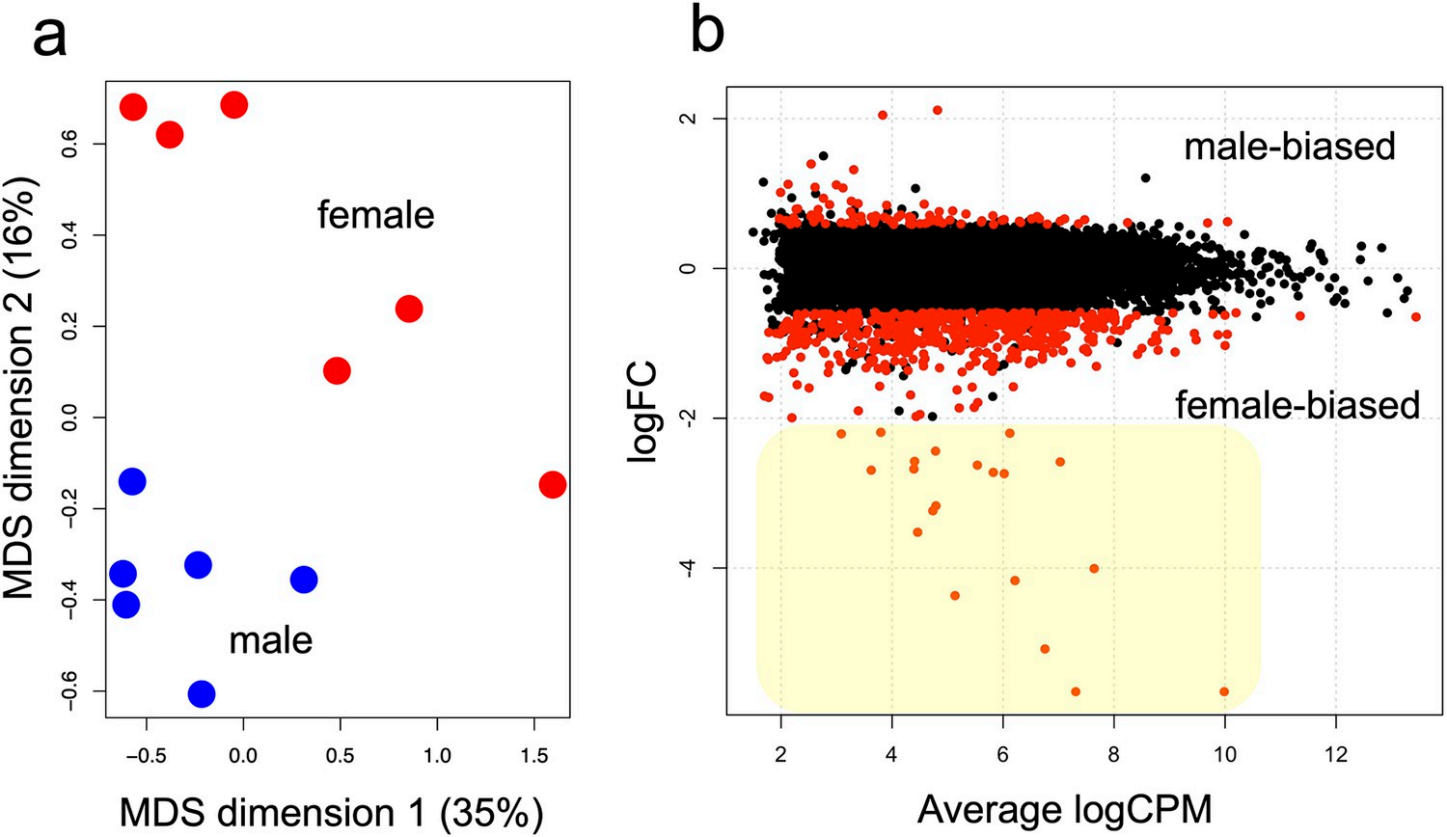
Comparative transcriptome shows the predominance of female-biased genes. a) Multidimensional scaling (MDS) plot of 12 samples shows transcriptomic difference of male (blue) and female (red) early prepupal head (n = 6 each). b) MA plot of comparative transcriptome. Among 20565 genes, 89 genes were male-biased and 584 genes were female-biased. Threshold of statistical significance (red dots) is FDR < 0.05 and expression changed >50% (|logFC| > log2(1.5)). Twenty genes in highlighted zone (yellow) were subjected to functional screening by RNAi.

One possibility is that the reduction of certain transcripts in males allows the acquisition of novel male structure. This may result in the relative increase of female-biased genes in male vs. female comparative transcriptome. Another, non-mutually exclusive possibility is that female-biased genes have functions to suppress the expression of male traits in females. This scenario is in concordance with the theoretical framework of sexually antagonistic selection and the resolution of sexual conflict [24,25]. If the acquisition of male weapon involves the modification of developmental gene regulatory network (GRN) in females, females should evolve to diminish the sexually antagonistic effect of novel GRN. Sexual dimorphism can be viewed as the consequence of a resolution of sexual conflict and therefore, female gene expression may also be modified to cope with the male trait evolution [24,25]. For these reasons, the modification of original GRN should not be limited to the increase of male-biased genes, but the significance of female-biased genes (i.e. apparently reduced transcripts in males) should also be visited. In this study, our comparative transcriptome of male and female prepupal head elucidated more abundant female-biased genes than male-biased genes (Figs 1 and 2, see result) in broad-horned flour beetle (*Gnatocerus cornutus*). This pattern motivated us to investigate the functional significance of female-biased genes.

Males of *G*. *cornutus* have exaggerated mandibles for male-male combat, and head horn and enlarged gena, likely used for display [26]. The goal of this study is to clarify the genes involved in the development of these sexually dimorphic traits. Comparative transcriptome approach is a powerful tool to screen the candidate genes but the causal relationship between differential expression and phenotypic outcome is rarely validated (but see [20,22]). Thus, the functional validation of the screened candidates should be a breakthrough in current eco-evo-devo research. In this study, RNAi-mediated functional validation of top 20 most sex-biased genes and 3 genes from top enriched GO (cell-cell adhesion) were performed in *G*. *cornutus*. Here we report that 4 of 23 genes functionally contributed the morphogenesis of exaggerated traits.

## Materials & methods

### Broad-horned flour beetle (*Gnatocerus cornutus*)

*G*. *cornutus* belongs to Tenebrioninae and is closely related to non-weaponed beetle genera such as *Tribolium* and *Neomida* [27]. Male mandible has bifurcated outgrowth structure that is called mandibular horn (mh, Fig 1A) and it is used for territorial combat [28,29]. Lateral region of male head (gena) is enlarged, and two head horns (hh) are located between eyes (Fig 1A). Male gena and head horn are likely used for display in combat [26]. Female lacks these structures (Fig 1B).

The stock population of *G*. *cornutus* originated in Miyazaki prefecture, Japan (31° 54′ N, 131° 25′ E) in June 1957. *G*. *cornutus* was reared in whole-wheat flour enriched with 5% of brewer’s yeast (EBIOS; Asahi Beer, Tokyo, Japan). In stock culture, approximately 200 of larvae were reared in group in a plastic container (65mm diameter, 105mm height) in 14L10D, 25°C.

### Genome sequence

*G*. *cornutus genome* was sequenced using a male from the stock culture. Genomic DNA extraction followed the sample preparation protocol (DNA extraction from single insects, 10x Genomics). DNA fragments shorter than 40kb was removed by Short read eliminator kit XL (Circulomics) and 0.673ng of DNA was subjected to Gel Bead-IN-EMulsion, Chromium linked read method following the instruction of Genome Reagent Kits v2 User Guide (10x Genomics). The sequencing was performed using TruSeq DNA Nano LT Library Prep kit 24 and HiSeqX (PE150, Illumina). Assemble was performed by supernova ver. 2.1.1 (10x Genomics). For annotation, Braker 2 was applied and Hisat2 [30] alignment files from transcriptomic samples were used to train Augustus.

### Comparative transcriptome between sexes

Prepupa of *G*. *cornutus* is available by isolation of final instar larva from group-rearing stock culture to solitary unfed condition. After isolation larvae develop into prepupae in 3 to 4 days and the subsequent prepupal period is approximately 2 days [31]. Prepupation is detected by its characteristic L-shaped posture. We collected early prepupae (day-1 prepupae, 0–24 hours after prepupation), and total RNA was extracted from their heads by RNA aqueous-Micro kit (Thermo Fisher). Individual RNA sample was reverse transcribed by Super Script II (Invitrogen) with PolyT START primer and subjected to the following library preparation [32]. For prepupal sex identification, individual cDNA was subjected to PCR amplification of the sex-specific isoforms of *doublesex* (*dsx*) in which males and females are distinguished by different amplicon sizes [18]. Primer sequences were (F: TATAGACCCGCATGTCCTGCAGA, R: GCAGAAGTCTAGGAGGATCTCGG). cDNA from 5 individuals (males or females) were pooled as one sample, and 6 biological replicates were performed.

The cDNA library preparation for RNA-seq followed the template switching method described in [32]. cDNA was subjected to NEBNext Ultra Directional RNA Library Prep Kit for Illumina and NEBNext Multiplex Oligos for Illumina, and DNA fragment size of 350-700bp was selected (Agencourt AMPure XP, Life Technoliogies; Agilent Bioanalyzer 2100, Agilent Technologies). The library sequencing was performed with Hi-seq 2000 (Illumina) provided by BGI Japan. The adapter sequences and low quality reads (Q<20) were removed with Cutadapt 3.4 [33], and the reads were mapped to *G*. *cornutus* genome by Hisat2 [30] and StringTie [34]. Gene-wise count data was analyzed with edgeR 3.40 [35]. DEGs were defined as FDR < 0.05 and |logFC| > log2(1.5) (i.e. >50% of expression change), and obtained DEGs were subjected to Gene Ontology (GO) enrichment analysis to detect the frequently observed GO terms in DEG lists compared to non-DEGs, using the code implemented in Omicsbox 3.1.2 (BioBam Bioinformatics).

### Functional screening by larval RNAi

For the screening of genes involved in development of exaggerated traits (mandible, head horn and gena), top 20 sex-biased genes were selected based on logFC (all were female-biased genes), and 3 genes from the most enriched GO (cell-cell adhesion; *fat 1/2/3*, *fat4*, *dachsous*) were selected (S1 Table).

Target genes were amplified with gene-specific primers with T7 adaptor sequence (primer sequences summarized in S1 Table). The PCR condition was 94°C, 5min, followed by 35 cycles of 94°C, 30 sec, 55–70°C (gradient), 30 sec, 72°C, 30sec, and terminated with 72°C 7 min (Takara ExTaq, Takara, Japan). We chose the appropriate annealing temperature from gradient annealing temperatures that yields the single band of predicted fragment size by agarose gel electrophoresis (AGE). PCR products were purified by ethanol precipitation, and subjected to Sanger sequence (Eurofin Genomics, Japan) to confirm the target sequence. Using these PCR products as templates, dsRNA was synthesized and purified by Ampliscribe-t7-flash-transcription-kit (Lucigen, US), following the manufacturer’s instruction. Fragment size and final amount of dsRNA was checked by AGE and Nanodrop One (Thermo Fisher, Japan).

Final instar larvae were randomly selected from the stock, and 50ng of dsRNA diluted in 4.6-138nl of TE buffer was injected to final instar larvae (n = 28–61), using Nanoject II (Drummond Scientific, US) under CO_2_ anesthesia. This method is known to reduce approximately 50% (37–85%) of target transcripts [13].For control individuals, 23nl of TE buffer was injected.

Obtained adults were firstly visually inspected for morphological effect of RNAi. Clear, qualitative phenotype was shown by photograph. When phenotypic effect is quantitative, morphological measurements were performed with digital microscope (DSX-WZHU, Olympus). In this study, prothorax width (PW) was used as body size index (covariable) to compare the trait size, because elytron is often malformed by gene knockdown (KD). Measured body parts were summarized in S1 Fig.

## Results

### *De novo* assembly and annotation of *G*. *cornutus* genome

As the basis of transcriptome and functional analysis of exaggerated traits, the genome of *G*. *cornutus* was sequenced using Chromium linked reads method. *G*.*cornutus* genome had a size of 150.3Mb with a scaffold N50 of 7.9Mb, a contig N50 of 128.5Kb and a total of 509 contigs (> = 10kb). Automatic annotation with Braker2, with manual addition of a previously described gene (gene ID: gm1, *ILP5*) yielded 22212 transcripts and 20565 genes. BUSCO analysis (ver. 4.1.2), based on insecta_odb10, showed 96.9% of complete, 1.3% of fragmented and 1.8% of missing gene models, implying that current genome covers most genes.

### Comparative transcriptome between sexes

Low count genes were filtered by keeping the genes that have at least 5 reads per million in at least 3 samples, and 9045 genes were remained. Multidimensional scaling plot confirmed that male and female samples have different transcriptomic profiles (Fig 2A). Comparison of genes expressed in early prepupal head revealed 673 sex-biased genes (Fig 2B, FDR < 0.05, |LFC| > log2(1.5), i.e. more than 50% of changes in gene expression was set as threshold, see S2 Table for gene list). Among these, 89 genes were male-biased and 584 genes were female-biased. Additionally, 168 genes had strongly negative logFC scores (< -1) whereas only 13 genes had strongly positive log FC scores (>1). Such predominance of female-biased genes motivated us for the functional screening of these strongly female-biased genes.

GO enrichment analysis for sex-biased genes elucidated 45 significantly enriched GOs and among them, GOs related with cell adhesion was frequently observed (Table 1, bold letters and S3 Table for full list). The top GO (GO:0098609), cell-cell adhesion included 15 genes and all of them were female-biased (Table 2). Interestingly, these genes included large cadherin molecules *fat* and *dachsous* (*ds*) that is necessary to establish planer cell polarity and regulate tissue growth by controlling the cell division direction [36,37]. Given that *fat* and *ds* are known to regulate the growth of beetle horn and stag beetle mandible [14,38], we focused these genes as the candidate weapon-morphogenetic genes and subjected to functional screening in *G*. *cornutus*. Before functional analysis, we checked the *fat* and *ds* sequences to confirm the number of gene copies by confirming the alignments with *Tribolium castaneum* orthologs. During this process, we found that two sequences encoding *ds* (jg2766 and jg2744) originates from a single gene but has been split by gene prediction error. Therefore, we used jg2774 as the target sequence of *ds*. *fat* genes had two copies, that are referred to as *fat1/2/3* and *fat 4*, following the annotation in *T*. *castaneum* (numbers correspond to mammalian homologs of fat1-4).

**Table 1:** Top 10 Enriched GO terms in sex-biased genes.

GO ID	GO Name	GO Category	FDR	P-Value	Genes in DEGs
GO:0098609	**cell-cell adhesion**	BIOLOGICAL_PROCESS	9.56E-06	1.75E-09	15
GO:0007155	**cell adhesion**	BIOLOGICAL_PROCESS	2.00E-05	7.32E-09	21
GO:0050794	regulation of cellular process	BIOLOGICAL_PROCESS	3.57E-04	4.14E-07	126
GO:0098742	**cell-cell adhesion via plasma-membrane adhesion molecules**	BIOLOGICAL_PROCESS	3.57E-04	4.72E-07	9
GO:0007156	**homophilic cell adhesion via plasma membrane adhesion molecules**	BIOLOGICAL_PROCESS	3.57E-04	4.72E-07	9
GO:1903506	regulation of nucleic acid-templated transcription	BIOLOGICAL_PROCESS	3.57E-04	5.22E-07	63
GO:0006355	regulation of DNA-templated transcription	BIOLOGICAL_PROCESS	3.57E-04	5.22E-07	63
GO:2001141	regulation of RNA biosynthetic process	BIOLOGICAL_PROCESS	3.57E-04	5.22E-07	63
GO:0065007	biological regulation	BIOLOGICAL_PROCESS	4.04E-04	6.63E-07	137
GO:0050789	regulation of biological process	BIOLOGICAL_PROCESS	4.88E-04	1.07E-06	131

Cell adhesion related GO terms were shown in bold. Fisher’s exact probability test.

**Table 2:** Most enriched GO (cell-cell adhesion) included 15 female-biased genes.

gene: annotation	logFC	logCPM	P-Value	FDR
**jg2766: protein dachsous**	-0.797	5.418	0.000	0.00
**jg1579: cadherin-related tumor suppressor (fat4)**	-0.953	7.005	0.000	0.000
jg15590: Calsyntenin-1-like Protein	-0.635	11.353	0.000	0.001
jg19870: Cadherin-related tumor suppressor-like Protein	-0.729	2.765	0.004	0.024
jg18789: I-set domain containing protein	-1.051	6.687	0.000	0.004
jg5183: neuroligin-1 isoform X1	-0.751	2.951	0.000	0.004
jg4130: neural-cadherin isoform X10	-0.761	5.129	0.001	0.007
jg224: I-set domain containing protein	-1.054	6.692	0.000	0.004
jg12279: PREDICTED: hemicentin-2	-0.741	2.742	0.001	0.009
jg4136: neural-cadherin isoform X9	-1.622	5.171	0.000	0.000
jg11159: protocadherin 15	-0.821	8.004	0.000	0.000
**jg2774: Protein dachsous-like Protein**	-0.939	4.252	0.000	0.000
**jg9117: fat-like cadherin-related tumor suppressor homolog isoform X1 (fat1/2/3)**	-0.956	6.032	0.000	0.000
jg973: neuroligin-4, X-linked	-0.672	2.901	0.003	0.018
jg5672: neuroligin-4, X-linked	-0.625	2.618	0.006	0.030

Negative logFC values show expression bias to female.

### Functional screening of weapon morphogenetic genes

Based on the log-fold change (LFC) values, top 20 sex-biased genes were all proven to be female-biased (Fig 2 and S1 Table). Therefore, top 20 female-biased genes (i.e. lowest logFCs), and 3 genes from most enriched GO (cell-cell adhesion; *fat 1/2/3*, *fat4*, *dachsous*) were selected as the targets of functional screening (Table 3). Among the 23 genes knocked down, 4 genes exhibited adult morphogenetic effects (Table 3). For all target genes, more than half of the RNAi treated beetles survived to pharate adults, but phenotypic effects were not detected for remaining 19 genes (Table 3).

**Table 3:** Effects of functional screening for exaggerated traits.

gene ID	annotation	criteria	logFC	survival (n)	phenotype
jg17521	Chitin bind 4 domain containing protein	top DEG1	-8.86	81% (n = 48)	-
jg8066	larval cuticle protein A3A-like	top DEG2	-6.28	41% (n = 59)	-
jg8067	cuticular protein CP3	top DEG3	-5.08	58% (n = 50)	-
jg17509	skin secretory protein xP2	top DEG4	-4.37	86% (n = 49)	-
jg11329	Chorion peroxidase-like Protein	top DEG5	-4.17	73% (n = 48)	-
jg8591	cuticular protein precursor	top DEG6	-4.01	77% (n = 48)	-
jg12786	PREDICTED: uncharacterized protein LOC664098	top DEG7	-3.52	59% (n = 46)	-
jg2242	tetra-peptide repeat homeobox protein 1-like	top DEG8	-3.24	84% (n = 51)	-
jg8492	Protein takeout-like Protein	top DEG9	-3.17	85% (n = 48)	-
jg18630	alpha-protein kinase 1	top DEG10	-2.74	96% (n = 48)	-
jg4861	putative fatty acyl-CoA reductase CG5065	top DEG11	-2.72	90% (n = 48)	-
jg9480	skin secretory protein xP2-like	top DEG12	-2.69	45% (n = 53)	-
jg390	cytochrome P450-like protein	top DEG13	-2.68	67% (n = 61)	-
jg4761	PREDICTED: uncharacterized protein LOC657853	top DEG14	-2.63	78% (n = 49)	-
jg2231	tetra-peptide repeat homeobox protein 1-like	top DEG15	-2.58	52% (n = 46)	-
jg5550	general odorant-binding protein 70	top DEG16	-2.58	77% (n = 48)	-
jg6431	PREDICTED: uncharacterized protein LOC103314049	top DEG17	-2.44	90% (n = 48)	-
jg16819	zinc finger protein 608-like	top DEG18	-2.21	76% (n = 45)	female gena enlargement
jg14513	alpha-tocopherol transfer protein-like	top DEG19	-2.20	47% (n = 45)	-
jg18813	long-chain fatty acid transport protein 4	top DEG20	-2.19	75% (n = 28)	-
jg1579	fat 4	top GO	-0.95	81% (n = 42)	male mandibular horn thick & short
					female vestigial mandibular horn
jg9117	fat 1/2/3	top GO	-0.96	52% (n = 46)	male head horn thick
jg2774	dachsous	top GO	-0.94	72% (n = 46)	male mandibular horn thick & short

Hyfen (-) means no detectable phenotypic effect.

**Fig 3:**
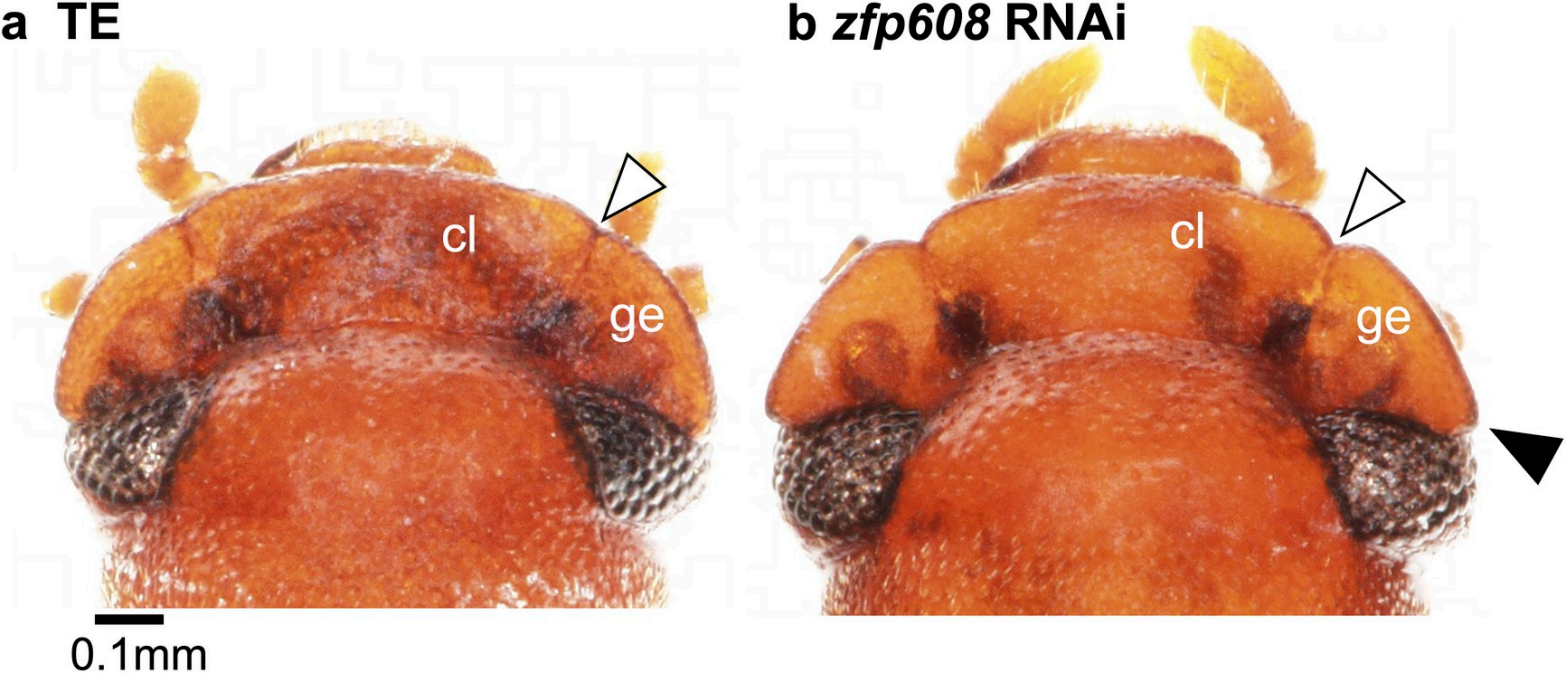
*zfp608* ^RNAi^ female head exhibited male-like gena structure. a) In normal female (TE), clypeus(cl) and gena (ge) were fused to form a fan-like head structure. Sclerites are only distinguished by striation (white arrow head). b) In *zfp608* ^RNAi^ female, gena is detached from clypeus (white arrowhead) and laterally protruded to form male-like structure (black arrowhead). This phenotype was observed in all *zfp608* ^RNAi^ females (n = 18).

Knockdown of one transcription factor, *zinc finger protein 608-like* (*zfp608*), caused a clear morphological change in female head (Fig 3). In normal female (Fig 3A), the clypeus and gena were fused and sclerites are distinguished only by striation (white arrowhead). In contrast, the gena of *zfp608*^RNAi^ female is detached from clypeus (white arrowhead) and laterally protruded (black arrowhead). This phenotype resembled male gena structure that is clearly detached from clypeus. No detectable effect was observed in *zfp608*^RNAi^ males.

**Fig 4:**
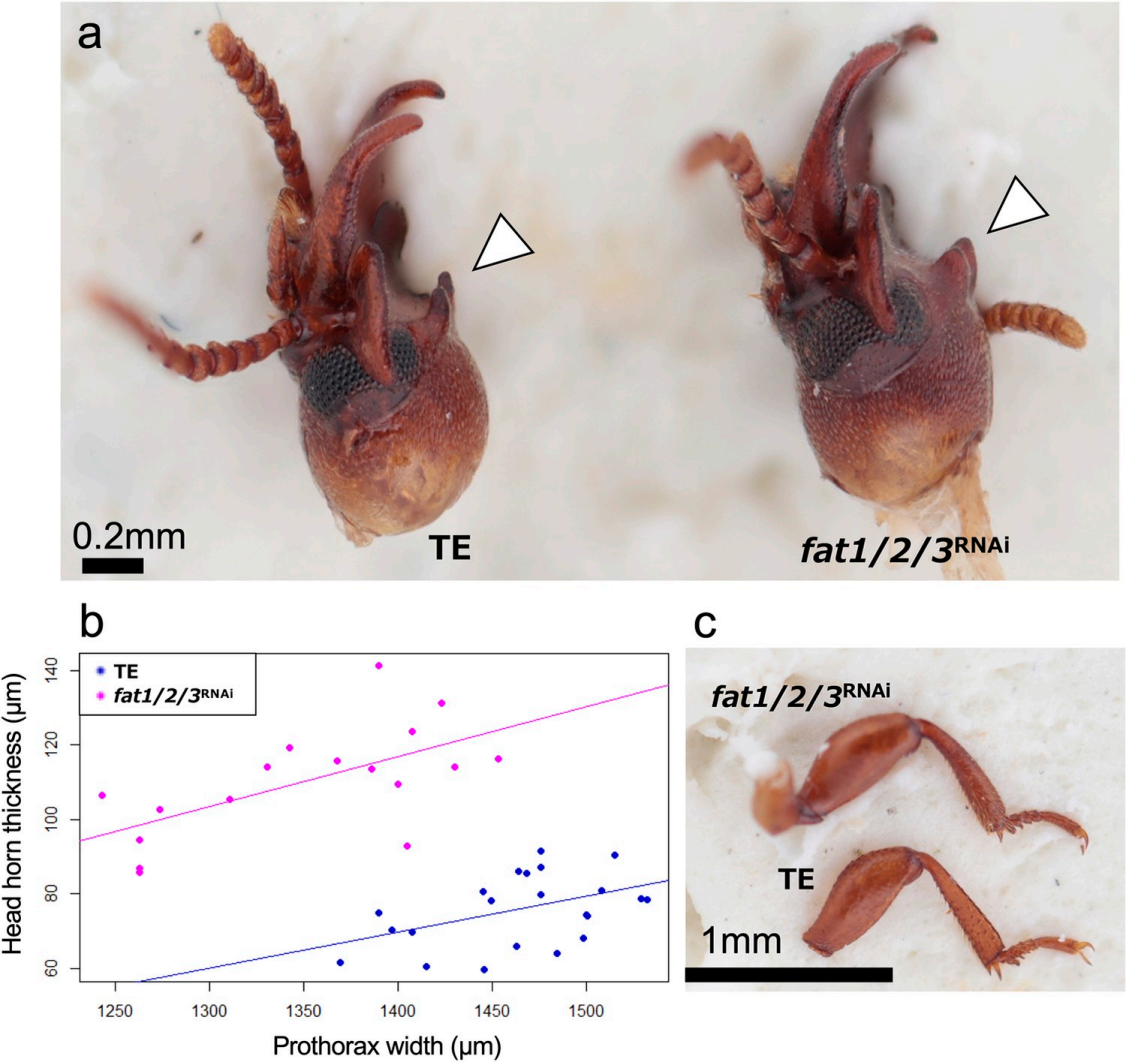
fat1/2/3 ^RNAi^ males had thicker head horn. a) Lateral view of normal (TE) and *fat1/2/3* ^RNAi^ male head. Head horn (white arrowheads) became thicker in *fat1/2/3* ^RNAi^ male. b) Morphological measurement shows head horn thickness is increased in in *fat1/2/3* ^RNAi^ males (ANCOVA: treatment, F = 104.9957, p < 0.001). Phenotypic effects on legs were not detected. Male foreleg is shown.

Knockdown of *fat1/2/3* reduced male head horn thickness (Fig 4A and 4B, ANCOVA: treatment, *F* = 104.9957, p < 0.001). In *fat1/2/3*^*RNAi*^ males, legs and other appendages were not affected (Fig 3C), and no detectable phenotype was observed in females.

**Fig 5:**
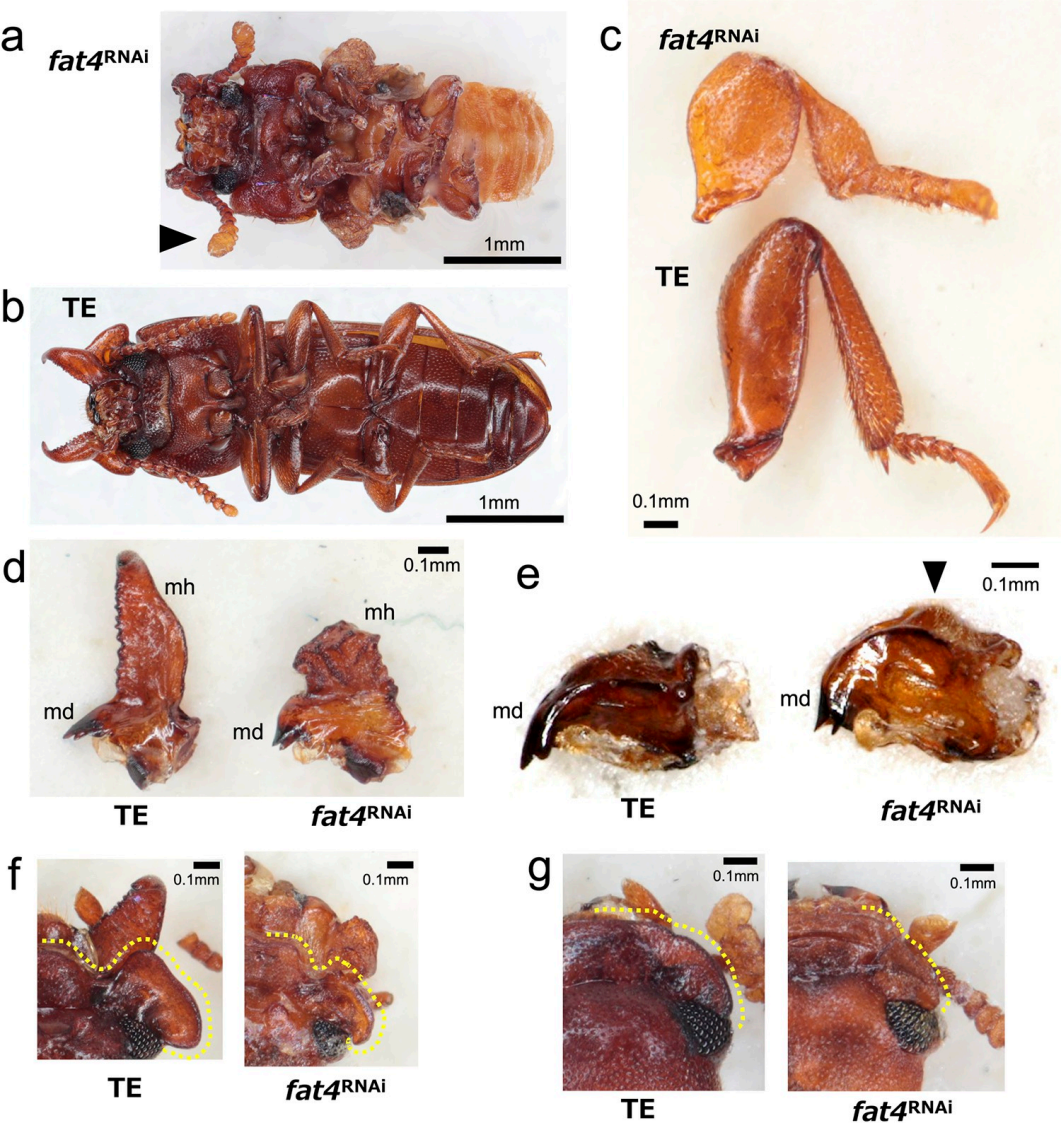
*fat4* ^RNAi^ phenotypes in male and female. a,b) *fat4* ^RNAi^ caused a severe systemic effect (ventral view). Antenna became short and thick (a, arrowhead). c) all legs were shortened and tarsomeres were fused in *fat4* ^RNAi^ adults. Male foreleg is shown. d) In *fat4* ^RNAi^ male, mandibular horn (mh) was malformed to show thick and short, rectangular column-like morphology. Mandible (md) became thick and short. e) In in *fat4* ^RNAi^ female, a bump was abnormally formed at outer ridge of mandible (arrowhead) where male mandibular horn develops in corresponding region. f) Gena was clearly reduced in male and slightly reduced in female (g). Dashed lines indicate the edge of gena and clypeus (f, g).

Knockdown of *fat4* had a severe systemic effect (Fig 5A and 5B). In both sex, antennae, mouthparts and legs were shortened (Fig 5A). Leg tarsomeres were fused in *fat4* ^RNAi^ adults (Fig 5B). In in *fat4* ^RNAi^ male, mandibular horn became thick and short, and was deformed to show rectangular column-like form (Fig 5D). Interestingly, in *fat4*^RNAi^ female, a bump was abnormally formed at outer ridge of mandible (white arrowhead) where mandibular horn develops in the male corresponding region. Both in *fat4*^RNAi^ males and females, mandibles became thicker and shorter (Fig 5D and 5E). Additionally, in *fat4*^RNAi^ individuals, gena size was clearly reduced in males (Fig 5F), and slightly reduced in females (Fig 5G).

**Fig 6:**
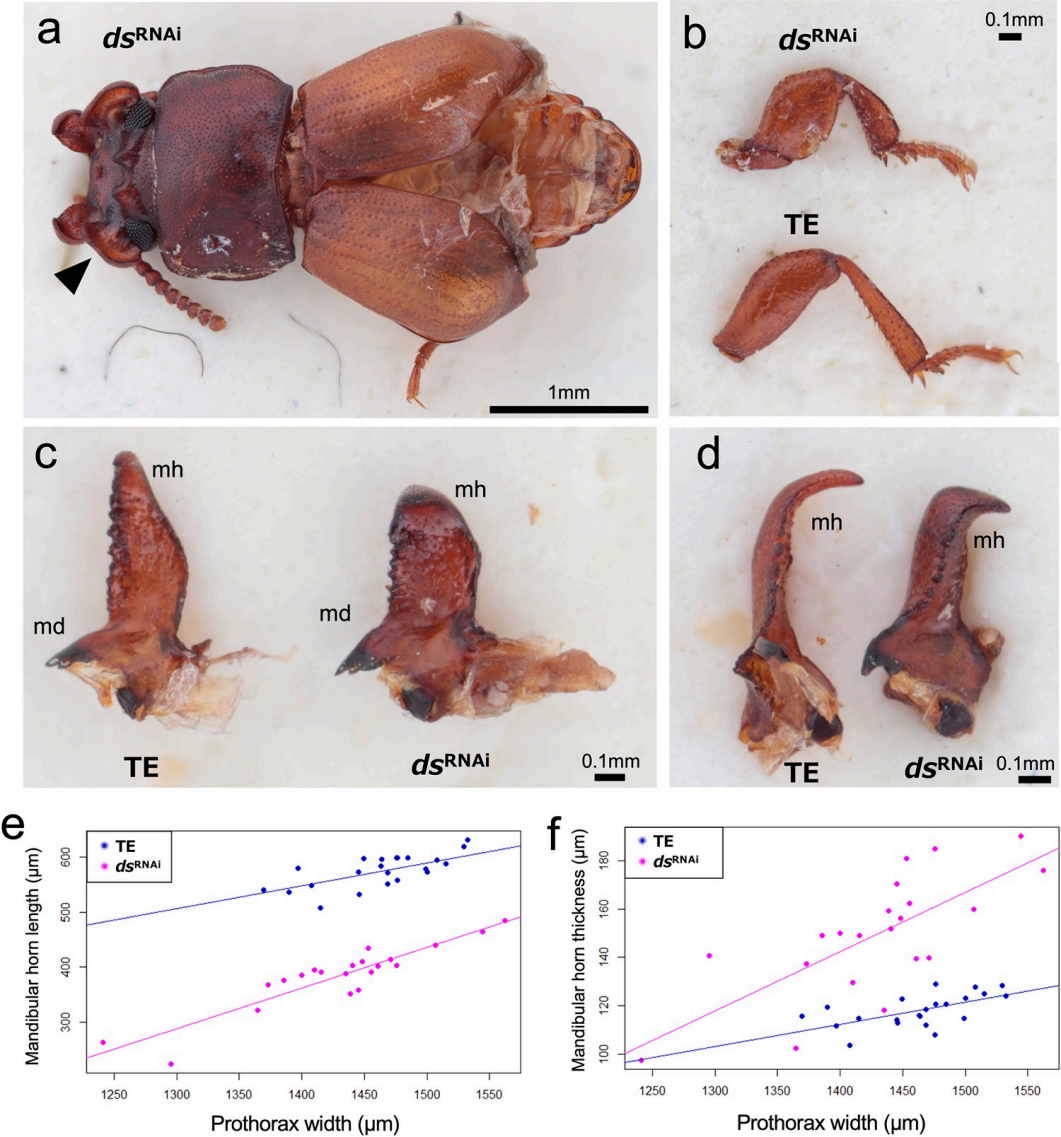
*dachsous*^RNAi^ males exhibited thick and short mandibular horns. a) Dorsal view of *dachsous*^RNAi^ (*ds*^RNAi^) male. Unlike *fat4*^RNAi^, antenna was unaffected. Gena ridge curled upwards and gena size was reduced (arrowhead). Elytra extension was incomplete. b) *ds*^RNAi^ adults showed short and thick legs but tarsomere fusion was not observed. Male foreleg is shown. c) Dorsal view of male right mandible. Mandibular horn (mh) was shortened in *ds*^RNAi^ males (e, ANCOVA: treatment, F = 8.0793, p < 0.01). d) Lateral view of male right mandible. *ds*^RNAi^ males had thicker mandibular horn (mh) (f, ANCOVA: PW×treatment, F = 4.7988, p = 0.03).

Knockdown of *dachsous* (*ds*) also induced short and thick legs (Fig 6A–6D), however, appendage-shortening effects were milder than that of *fat4*^RNAi^ in lacking antennal effect and tarsomere fusion. In *ds*^RNAi^ males, mandibular horn became shorter (Fig 6C and 6E, ANCOVA: treatment, *F* = 8.0793, p < 0.01) and thicker (Fig 6D and 6F, ANCOVA: PW×treatment, *F* = 4.7988, p = 0.0345). Gena ridge curled upwards and gena size was reduced in *ds*^RNAi^ males (Fig 6A, arrowhead). No apparent effects on mandible and gena were detected in *ds*^RNAi^ females.

## Discussion

### Comparative transcriptome between sexes

By focusing on the sex-biased transcripts expressed during adult head morphogenesis (i.e. early prepupa), this study identified 673 sex-biased genes as candidates for weapon-morphogenetic genes. Since *G*. *cornutus* is closely related to non-weaponed beetle genus *Tribolium* and *Neomida* [27], exaggerated male traits of *G*. *cornutus* are considered to be derived novel traits, and female *G*. *cornutus* resembles ancestral phenotype of sister groups. Intuitively, the acquisition of male weapon, i.e. the increase of morphological complexity is expected to be associated with the increase of male-biased genes. However, our comparative transcriptome between sexes revealed that female-biased genes (584 genes) were predominant in sex-biased genes. Such predominance of female-biased genes in male-exaggerated trait is also known in the head horn of rhinoceros beetle and the third leg of water strider during development [20,21]. Assuming that the gene regulatory network (GRN) of female development resembles that of weapon-less ancestor, it is possible that the acquisition of male weapon can be associated with the reduction of some transcripts from ancestral transcriptomic pattern. Our data, together with above examples, imply the overlooked significance of female-biased genes in weapon development and evolution.

The actual developmental functions of female-biased genes were investigated through RNAi-mediated functional screening of 23 genes. The knockdown of one transcription factor, *zinc finger protein 608-like* (*zfp608*) yielded the female with masculinized gena (i.e. lateral protrusion and detachment from clypeus, Fig 3). This means *zfp608* suppresses gena overgrowth in normal females. However, no effect was detected in *zfp608*^RNAi^ males, probably due to that males naturally have sufficiently low *zfp608* transcripts for gena overgrowth. In *Drosophila*, there is one *zfp608* homolog, called *brakeless* (also known as *scribbler* or *master of thickveins*) and this gene mainly function as a transcriptional co-repressor, working with Atrophin and Tailless. During development, Brakeless affects embryonic segmentation, and adult wing and brain morphogenesis by altering many downstream genes [39–41]. Although head developmental function of this gene had not been reported, *zfp608* may suppress downstream genes regulating gena morphogenesis in *G*. *cornutus*.

### Cell adhesion molecules affect outgrowth morphogenesis

GO enrichment analysis of sex-biased genes elucidated the frequent female-biased expression of cell adhesion molecules, implying the sex difference of cell adhesion process in head development. Interestingly, the most enriched GO term (i.e. cell-cell adhesion) included two *fat* genes (*fat1/2/3*, *fat4*) and one *dachsous* (*ds*) gene. Importantly, these genes are known to regulate the elongation of appendages in fruit fly [42] and weapons in Scarabaeidae beetles; *fat* and *ds* deficiency caused shortening of male mandibles and horns [14,38]. These transmembrane proteins are fundamental for the establishment of planer cell polarity (PCP) during imaginal disc growth [36]. Fat and Ds proteins are known to form heterodimers and the heterodimer is more abundant in proximal region than in distal region in the growing tissue. Such gradient distribution of active proteins coordinates the direction of cell division and morphogenesis [36,43]. In horned beetle, KD of *fat* and *ds* caused the development of shorter and thicker horn than non-treated beetles and this is caused by a change in cell division direction from proximal to distal [14,43].

*fat4*^RNAi^ and *d*s^RNAi^ males and females showed thick, shortened legs (Figs 5C and 6B), the common characteristic phenotypes of *fat* and *ds* deficiencies in fruit fly, cricket and beetles [14,38,42,44]. The tarsomere fusion in *fat4*^RNAi^ adults (Fig 5C) also confirms functional commonality with other insects [14,42]. Therefore, the appendage elongation function of *fat4* and *ds* is highly conserved in *G*. *cornutus* and other insects. In *G*. *cornutus*, gena became smaller (Figs 5F and 6A) and mandibular horn became thick and short (Figs 5D, 6C and 6D) in *fat4*^RNAi^ and *d*s^RNAi^ males. Additionally, *fat1/2/3*^RNAi^ males had thicker head horn, though length difference was unclear due to the ambiguous horn structure in *fat1/2/3*^RNAi^ males (Fig 4A and 4B). From these phenotypic effects, we conclude that the conserved appendage elongation functions of *fat* and *ds* are likely to be co-opted for the formation of these male outgrowth structures in broad-horned flour beetle.

Notably, *fat4*^RNAi^ female formed a horn-like bump on mandible (Fig 5E). This abnormal formation of rudimentary male-like structure suggests that *fat4* normally suppress mandibular horn expression in females. However, *fat4*^RNAi^ male had a malformed, short and thick mandibular horn lacking the apical structure. Therefore, the suppression of mandibular horn by *fat4* is inconsistent across sexes. Given that female mandibular structure is likely ancestral, the *de novo* formation of mandibular horn may require a developmental modification from ancestral state. We speculate that reduction of *fat4* expression may allow the formation of novel outgrowth on mandibles via changes in planer cell polarity and cell division direction. However, the formation of fully elongated male structure may require the appropriate amount of *fat4* expression. The future study will need to clarify the localization of fat4 in mandible to understand how this gene is deployed in mandibular horn development.

So far, the weapon morphogenetic effect of *fat* and *ds* had been known for horns of rhinoceros beetle and the mandibles of stag beetle [14,38] that are phylogenetically distant from Tenebrionidae [45]. Our study highlighted that these common genes were independently deployed in outgrowth acquisition in *G*. *cornutus*. Morphologically, the mandibular horn of male *G*. *cornutus* is a novel branching outgrowth on the outer ridge of a mandible [29], whereas the long mandible of male stag beetle is the elongation of an existing mandible itself [6] and the horn of rhinoceros beetle is a novel outgrowth on head [20]. Therefore, the morphological origins of these weapons are also independent. Taken together, the modification of cell adhesion process and planar cell polarity may have enabled the acquisition of outgrowth in multiple taxa, and *fat* and *ds* are repeatedly deployed key genes in the evolution of beetle weapons.

### Implication for evolution of sexual dimorphism

The sexually selected weapons and ornaments provide mating advantages to males, but are costly if expressed in females. Sexually selected exaggerated traits generally only develop in males i.e., sexual dimorphism [1–3], and this is considered as a consequence of the resolution of sexual conflict over trait expression [24,25]. Our study illustrated that some of female-biased genes actually suppress the expression of male-exaggerated traits in females, shedding light on the significance of female-biased genes in the evolution of sexually dimorphic exaggerated traits. The future comparative analysis of GRN in weapon-less sister species (e.g. red flour beetle) may uncover the evolutionary significance of female-biased genes in sexually antagonistic trait evolution.

## Supporting information

S1 Table Enriched GOs in DEGs. (XLSX)

S2 Table Full list of genes for comparative transcriptome. (CSV)

S3 Table Full list of enriched GOs. (XLSX)

S1 Fig Measurement of body parts. Measurement of body parts. a) Prothorax width. Maximum width of prothorax. b) Head horn length. In vertical view, distance from the striation at the root of horn to its tip was measured. c) Head horn thickness. In lateral view, the intersection of horn and head capsule was set as a landmark, then the direction vertical to the horn protrusion was measured. d) Mandibular horn length. In ventral view, intersection of feeding part of mandible and mandibular horn was set as a landmark, and the distance to the tip of mandible was measured. e) Head horn thickness. Left mandible was viewed from inner lateral side widest part was measured.
(PDF)
