# Supplementary material for: Transcriptomic and functional screening of weapon formation genes implies significance of cell adhesion molecules and female-biased genes in broad-horned flour beetle
Source: PLoS Genet. 2023 Dec 5;19(12):e1011069. doi: 10.1371/journal.pgen.1011069 (PMC10723671; doi:10.1371/journal.pgen.1011069)
Supplement: S1 Fig — Measurement of body parts. a) Prothorax width. Maximum width of prothorax. b) Head horn length. In vertical view, distance from the striation at the root of horn to its tip was measured. c) Head horn thickness. In lateral view, the intersection of horn and head capsule was set as a landmark, then the direction vertical to the horn protrusion was measured. d) Mandibular horn length. In ventral view, intersection of feeding part of mandible and mandibular horn was set as a landmark, and the distance to the tip of mandible was measured. e) Head horn thickness. Left mandible was viewed from inner lateral side widest part was measured. (PDF) [file pgen.1011069.s004.pdf]

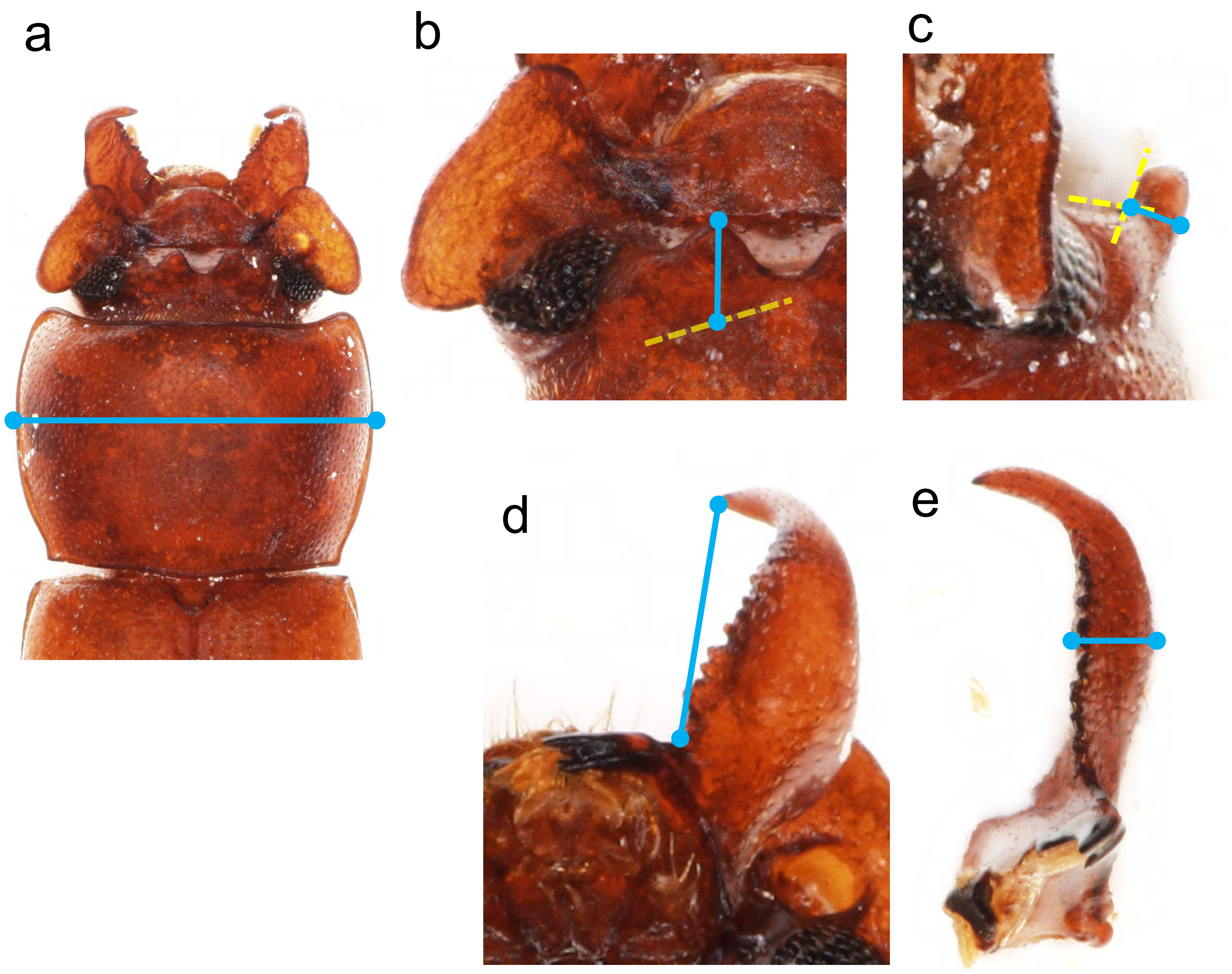

Fig. S1. Measurement of body parts

a) Prothorax width. Maximum width of prothorax. b) Head horn length. In vertical view, distance from the striation at the root of horn to its tip was measured. c) Head horn thickness. In lateral view, the intersection of horn and head capsule was set as a landmark, then the direction vertical to the horn protrusion was measured. d) Mandibular horn length. In ventral view, intersection of feeding part of mandible and mandibular horn was set as a landmark, and the distance to the tip of mandible was measured. e) Head horn thickness. Left mandible was viewed from inner lateral side widest part was measured.
